# Interspecific social networks promote information transmission in wild songbirds

**DOI:** 10.1098/rspb.2014.2804

**Published:** 2015-03-22

**Authors:** Damien R. Farine, Lucy M. Aplin, Ben C. Sheldon, William Hoppitt

**Affiliations:** 1 Department of Zoology, Edward Grey Institute of Field Ornithology, University of Oxford, Oxford OX1 3PS, UK; 2 Research School of Biology, Australian National University, Acton, Australian Capital Territory 2000, Australia; 3 Animal and Environment Research Group, Anglia Ruskin University, Cambridge CB1 1PT, UK

**Keywords:** mixed-species flocking, network-based diffusion analysis, public information, social information, social networks, transmission networks

## Abstract

Understanding the functional links between social structure and population processes is a central aim of evolutionary ecology. Multiple types of interactions can be represented by networks drawn for the same population, such as kinship, dominance or affiliative networks, but the relative importance of alternative networks in modulating population processes may not be clear. We illustrate this problem, and a solution, by developing a framework for testing the importance of different types of association in facilitating the transmission of information. We apply this framework to experimental data from wild songbirds that form mixed-species flocks, recording the arrival (patch discovery) of individuals to novel foraging sites. We tested whether intraspecific and interspecific social networks predicted the spread of information about novel food sites, and found that both contributed to transmission. The likelihood of acquiring information per unit of connection to knowledgeable individuals increased 22-fold for conspecifics, and 12-fold for heterospecifics. We also found that species varied in how much information they produced, suggesting that some species play a keystone role in winter foraging flocks. More generally, these analyses demonstrate that this method provides a powerful approach, using social networks to quantify the relative transmission rates across different social relationships.

## 1. Introduction

Social information is important for the ecology of many animal species. Observing others can provide naive individuals with diverse information, ranging from habitat quality and predator presence, to mate choice [1–3]. If individuals vary in their access to information, or if information spreads non-randomly between dyads, then population structure may play a crucial role in mediating the spread of information [4]. However, individuals will often have a choice of information sources, for example they can choose to observe conspecifics and/or heterospecifics. While social information from conspecifics may be the most relevant, relying solely on information from conspecifics can also lead to increased competition for limited resources, such as food or territories [3]. An alternative, or complementary, strategy might be to acquire information from heterospecifics. For example, many species eavesdrop on heterospecific alarm calls [5], and migrating birds preferentially copy the habitat choices of resident heterospecifics [6]. Here, we address the general problem of identifying the contribution of different types of associations to population processes, where different types of relationships form different social networks within the same set of individuals.

In a recent study [7], we experimentally tested whether information was transferred through social networks of wild songbirds. In that study, we used automated techniques to map association patterns in wild mixed-species flocks of tits (*Paridae*). We then experimentally deployed novel foraging patches and tracked the diffusion of information about these ephemeral food sources, finding that tits used social information from their associates (represented by edges in the social network) to locate new foraging resources. However, our study included individuals from three species that form mixed-species flocks: blue tits (*Cyanistes caeruleus*), great tits (*Parus major*) and marsh tits (*Poecile palustris*) [8,9]. Existing analytical tools cannot discriminate between the potentially different pathways of information flow between and within species. That is, while we found that birds used social information to find food (the order and time of discovery was predicted by the presence of edges in the social network), we could not test whether individuals used information from both conspecific and heterospecific associates when searching for food. If individuals in mixed-species foraging groups did use information from heterospecifics, then it is also important to determine how much they weighed information from heterospecifics and whether this differed from conspecifics.

Studies of social learning and the diffusion of information have generally also assumed that each link in an individual's social network can provide information at an equal rate. Yet, animal groups may be structured by multiple types of social relationships [10,11], each representing a different set of network edges. While these are often combined into a single network, such relationships may differentially promote or restrict population processes. For example, VanderWaal *et al*. [12] compared a network of genetic subtype similarity of the microbe *Escherichia coli* between giraffes (*Giraffa camelopardalis*) with networks describing giraffe social associations and habitat use overlap. They found that the association network (co-occurrence in social groups) best matched the similarity in pathogens between individuals. By contrast, networks created based on how much each pair of individuals shared water resources or overlapped in their home-range did not reflect the pathogens that individuals shared. This suggests that population processes, in this case pathogen transmission, can be mediated by different components of social structure or types of relationships. However, their study, and similar studies of transmission networks [13–16], only estimate the correlation in the structural similarities between the pathogen and social networks. A powerful approach for understanding how population structure facilitates information (or pathogen) transfer is to experimentally seed a behavioural innovation and track its spread (or ‘diffusion’) through the social network. This approach can then be combined with statistical tests that can control for heterogeneity in ecological-, individual- and population-level factors.

Network-based diffusion analysis (NBDA) has become a widely used method for investigating information transmission dynamics in animal groups [7,17–21]. NBDA infers the rate of social transmission of information by comparing the diffusion of information with patterns of association in the social network [22]. It assumes that the rate at which social transmission occurs is proportional to the strength of association between naive and informed individuals. NBDA has thus far, to our knowledge, been applied on only two wild animal populations: our study [7,23], and once to track the spread of lobtail feeding behaviour in a population of humpback whales over 30 years [18]. In these cases, the studies took potential confounds into account, but only tested the spread of information on a single association network.

In this study, we extend the NBDA analytical framework to test whether the rate of information transfer differs for different types of relationships. In our case, this enables us to explicitly determine whether information concerning novel food patches was transmitted through both intra- and interspecific social networks in the data from Aplin *et al*. [7], and if the rate of transmission (or the propensity to use information) differed. This allows us to quantify the benefit of associating with heterospecifics in terms of information access, which is considered a fundamental driver of mixed-species communities [24]. Given the extensive niche overlap between blue tits, great tits and marsh tits, we predicted that individuals should be using at least some social information indiscriminately. That is, information about novel food patches should spread to heterospecifics without requiring independent (non-social) discovery events to happen in each of the species. The ability to determine the relative rates of transmission across a number of potential pathways is therefore a powerful analytical approach for investigating the contribution of social relationships in population processes.

## 2. Methods

### (a) Study area and population

The data for this study [7] were collected from two small areas of broadleaf deciduous woodland near Wytham Woods, Oxfordshire (51°46′ N, 1°20′ W) that form part of an on-going study on social behaviour in birds [25,26]. Here, blue tits *C. caeruleus*, great tits *Pa. major* and marsh tits *Po. palustris* form mixed-species flocks in the non-breeding season. Individuals were caught using mist-nets and fitted with passive integrated transponder (PIT) tags, allowing them to be detected by radiofrequency identification (RFID) antennae fitted to standard bird feeders (Francis Instruments Ltd., Cambridge). The field data were then collected in two phases. The first measured the association patterns of individuals to construct a social network for each site. The second deployed randomly placed novel food patches around the study site to record each individual's first arrival at the resource.

### (b) Inferring social networks

The social network was inferred from the co-observations of individuals visiting feeders at two fixed sites in each area. Feeders were filled with food for 3 days and left empty for 3 days. This cycle was repeated continuously from December 2010 to January 2011 at Cammoor/Stimpsons Copse, and during January 2011 at Higgins Copse. Data loggers recorded the 15 s time block of each individual's visit along with its unique PIT-tag code. Using the R package *asnipe* [27], we then inferred dyadic association strengths from the spatio-temporal co-occurrences between individuals. We defined a network edge as the proportion of time two individuals are observed together (calculated using the simple ratio index, SRI), where an absent edge (weight = 0) indicates that they were never co-observed, and an edge weight of 0.5 indicates that in half of the observations of the two birds, they were seen together. This provides an estimate of the proportion of time any two individuals A and B spend under the conditions defined to constitute ‘association’. As such this is likely to provide a good measure of the opportunities A has to learn things from B, and vice versa, so long as the definition of ‘association’ does so (see the definition of *s* in the NBDA model below). Simulations of these types of networks suggest that the SRI provides a robust estimate of the underlying association patterns if the individuals are sampled numerous times (on average, we detected each individual 139 times, well in excess of the guidelines provided by Franks *et al*. [28]). However, because the SRI can result in large edge weights for rarely observed individuals, we also include the number of observations to account for this potential confound (see below).

**Figure 1.**
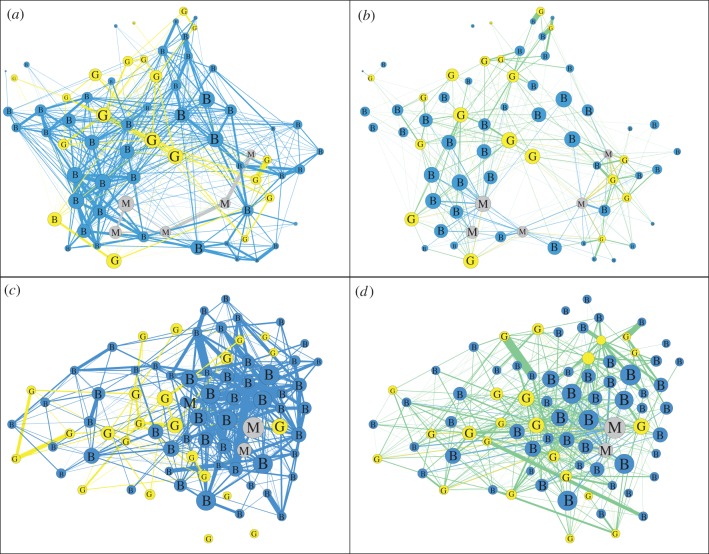
For each of the two areas in the study ((*a,b*) Cammoor/Simpsons Copse; (*c,d*) Higgins Copse) we formed two candidate social networks. One network contained all of the associations between conspecifics (*a,c*), with all the edges that were observed between nodes of the same species. The other network contained all of the associations between heterospecifics (*b,d*), with all the edges that were observed between nodes of different species. Node colour and label represents species (blue, B: blue tits; yellow, G: great tits; grey, M: marsh tits). Similarly, edge colour is the combination of the connecting nodes (e.g. green are edges between great tits and blue tits). Node size represents eigenvector centrality, which was calculated in the original study [7]. (Online version in colour.)

In the original study, we generated one mixed-species social network for each of the areas. In this study, we use the same edge definition, but split the network from each area into two subnetworks: one containing all edges that represent the association strengths between conspecifics, and a second network containing all edges between heterospecifics. Although the conspecific network contains three separate components (one for each species), it was retained as a single network (i.e. one association matrix) in our analysis (social networks do not need to be one fully connected component). This resulted in a total of four networks as shown in figure 1.

### (c) Patch discovery experiment

The patch discovery experiment began 14 days after the end of the network data collection, during which time no feeders were present and no supplementary food available. To record the order and time of arrival at new feeding locations, we placed a single feeder, equipped with RFID antennae in a random location in each area. Each feeder was removed after 3 days, and no food provided for 7 days between trials. This was repeated four times at Cammoor/Stimpsons Copse and three times at Higgins Copse. Previous analyses of the discovery events found that different individuals initially located each food patch, but that information was then socially transmitted, with the order of subsequent discoveries matching the patterns of association in the social network for each area (in which conspecific and heterospecific associations were combined) [7].

### (d) Multi-network network-based diffusion analysis

In the previous study, we used a single multi-species network from each site. This did not allow us to test whether social information was transmitted between heterospecifics, and if individuals relied more heavily on information from conspecifics. Information could have spread entirely between conspecifics (i.e. along conspecific network edges), requiring only three independent acquisitions (one individual per species discovering the food sources). The standard model of NBDA cannot determine whether transmission rates vary between links given observed social networks. Here, we extend the NBDA framework to include multiple candidate networks for each diffusion event, which in this case are the conspecific and heterospecific social networks described above (figure 1).

In the standard NBDA model, the rate at which individual *i* acquires information or adopts a novel behaviour at time *t* is given by [20,22]
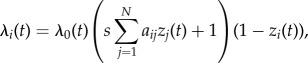
where *λ*_0_(*t*) is the baseline, or *asocial*, rate of acquisition (how fast individuals discover the information for themselves). In this study, we used the continuous time of acquisition diffusion analysis (continuous TADA) variant of NBDA [29]. This allows *λ*_0_(*t*) to be either constant (i.e. *λ*_0_(*t*) = *λ*_0_), or to increase or decrease systematically over the course of a diffusion. The *rate of social transmission* is given by parameter *s*. This parameter dictates the contribution of the weighted network edges (*a_ij_*) that connect *i* to informed individuals, given that *z_j_*(*t*) is the state of *i*'s social associates (0 = naive, 1 = informed). Because the *s* parameters give the rate of transmission per unit connection, when the SRI is used the *s* parameters effectively estimate the rate that social transmission occurs between two individuals when they are in association. Finally, the overall rate of acquisition *λ_i_*(*t*) is set to 0 once individual *i* changes to an informed state (1 − *z_i_*(*t*) = 0). Thus, if the social network has no predictive power, then *s* = 0 and the overall rate of acquisition will be given by the asocial rate of acquisition *λ_i_*(*t*) = *λ*_0_(*t*). Non-zero values of *s* suggest that individuals are associated with other informed individuals when they are observed to acquire the information or adopt the novel behaviour themselves.

To create a multi-network NBDA model, we expanded the standard NBDA model to include the effects of *M* different networks as follows:
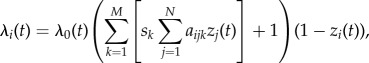
where *a_ijk_* is the network connection from *j* to *i* in network *k* and *sk* gives the rate of social transmission in network *k*. This same approach can be applied to any variant of NBDA (such as order of acquisition diffusion analysis), and further details are provided in the electronic supplementary material.

Because flocks of birds typically forage in groups, we wanted to isolate information transfer from co-discoveries by groups of birds. That is, our definition of information transfer should only include direct or indirect recruitment to the resource by knowledgeable birds. The simultaneous discovery by individuals in a flock should be considered more similar to food being found by individuals alone (defined by the asocial parameter *λ*_0_(*t*)). We did this by including an additional term in our model *T_ij_* to capture ties in the discoveries. We defined individuals (say A and B) that discovered food within 10 min of each other to be tied (*T_ij_* = 0), and ties are no longer included in the estimate of social information transfer (i.e. by setting *a_ij_kz_j_*(*t*)*T_ij_* = 0). However, if A and B did not discover the feeder together, and are connected in the social network, then social transmission may have occurred between them (*T_ij_* = 1). We consider this to be a conservative estimate of broader social information use, given that individuals may also be responding to social queues at a much finer scale [9].

The multi-network NBDA will work most effectively when the networks are independent. When they are highly dependent (e.g. correlated), it will require a lot of data to distinguish the effects of each network. This will be reflected in wide confidence intervals (CIs) for each *s* parameter, and for the estimated difference between them.

To infer the rates of transmission through the intraspecific and the interspecific social networks, we thus used
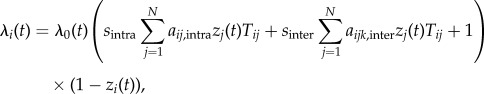
where *a_ij_*_,intra_ are the edge weights (association strengths) in the conspecific network, and *a_ij_*_,inter_ are the edge weights in the heterospecific network. Thus, if there is no transmission between heterospecifics (via the links in the relevant heterospecific network), then *s*_inter_ will equal 0. Finally, as with the standard NBDA model, it is also possible to incorporate linear predictors for each individual given by *V* variables in either a multiplicative or additive model. These are analogous to fixed effects in generalized linear models, and details are provided in the electronic supplementary material.

The best-fitting value of each parameter from the experimental diffusion data is then calculated by finding the maximum of the log-likelihood function (provided in [30]) using the optim function in *R*. This was done by calculating the log-likelihood for each of our seven diffusions independently, with the association strengths *a_ij_*_,intra_ and *a_ij_*_,inter_ taken from the social networks of the area where the diffusion took place (four in Cammoor/Stimpsons Copse and three in Higgins Copse). The total log-likelihood was the sum of the log-likelihoods over the seven diffusions, and parameter values that resulted in the largest sum were those that best fit our data.

### (e) Estimating rates of social and asocial acquisition of information

Our framework enabled us to estimate separate parameters *s*_intra_ and *s*_inter_ for the conspecific and heterospecific networks, giving the rate of social transmission per unit association in each of these networks independently. Our analysis included species as a factor (to allow for differences in discovery rate among species), and the number of observations of each bird from the social network data (to control for residency, per [7]). We fitted models with social transmission occurring: (i) at different rates within and between species (*s*_intra_ ≠ *s*_inter_); (ii) at the same rate (*s*_intra_ = *s*_inter_); (iii) only within species (*s*_inter_ = 0); (iv) only between species (*s*_intra_ = 0); (v) homogeneously between all individuals in a diffusion 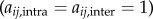, i.e. not following a specific social network; and (vi) with no social transmission (*s*_intra_ = *s*_inter_ = 0). Because diffusions occurred at two different sites, an indicator variable was included in all the models to allow the relative rate of discovery to differ at each site.

For each combination of variables, both additive and multiplicative models were fitted. In the additive model, each variable (fixed effect) is assumed to affect only the rate of asocial learning, with social transmission operating as an independent process by which patches could be discovered. In the multiplicative model, variables affect both the rate of asocial learning and social transmission. For example, if individual A is twice as fast to learn asocially as individual B, individual A will remain twice as fast as individual B if they have the same connection to informed individuals (see the electronic supplementary material for specifications). We used an information theoretic approach with corrected Akaike's information criterion (AIC_c_) to allow for model selection uncertainty, summing Akaike weights to get the level of support for hypotheses (i–vi), and obtaining model-averaged estimates for each parameter [31]. We obtained 95% CIs using the profile likelihood technique, conditional on the best model in which that parameter was present (full details are provided in the electronic supplementary material).

## 3. Results

**Figure 2.**
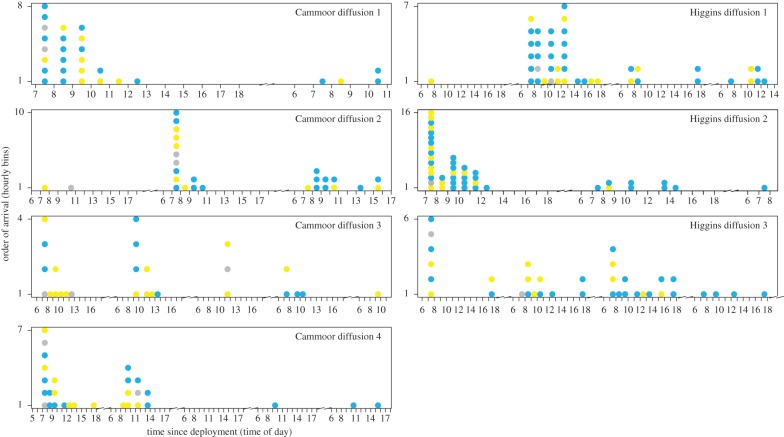
Arrival time and order for each experimental diffusion. Each diffusion was tested against the networks from that area (Cammoor/Stimpsons Copse with figure 1*a,b* and Higgins Copse with figure 1*c,d*) to estimate social and asocial rates of information acquisition. Each newly arrived individual is shown by a coloured point (blue: blue tit, yellow: great tit, grey: marsh tit). Arrival times were binned by hour, but the order of arrivals was maintained (from bottom to top). (Online version in colour.)

We recorded a total of 11 866 and 7790 feeding visits by 93 and 81 individuals, respectively, at Cammoor/Stimpsons Copse and Higgins Copse, respectively. From these data, we generated an intraspecific and an interspecific network for each area (figure 1). Blue tits were the most common species present, followed by great tits, whereas marsh tits were relatively uncommon (table 1). In total, 64% of birds in the network discovered at least one food patch (table 1), and only 11 birds (7%) were detected in both areas. We found that patterns of individual discoveries varied between diffusions, and exhibited isolated bursts of activity that are consistent with social information spread (figure 2). In our analysis, these individuals moving as a group and discovering patches together were considered to represent single cases of information transfer.

**Table 1.** Summary of individuals for each network and diffusion trial by area and by species. (Numbers represent how many individuals found the feeder in each diffusion (*individuals discovering*) and how many individuals were included in the social networks (*individuals in network*) of each area.)

area	diffusion	blue tits	great tits	marsh tits	total
Cammoor/Stimpsons	1	16	6	2	24
	2	12	4	3	19
	3	6	8	3	17
	4	14	7	3	24
*individuals discovering*		*28*	*11*	*4*	*43*
*individuals in network*		*51*	*25*	*5*	*81*
Higgins	1	25	11	2	37
	2	20	12	1	33
	3	12	7	2	21
*individuals discovering*		*38*	*15*	*2*	*55*
*individuals in network*		*66*	*25*	*2*	*93*
*total individuals discovering*		*68*	*30*	*5*	*103*
*total individuals in networks*		*106*	*49*	*7*	*162*

The addition of interspecific links in the social networks significantly changed the structure of the social network. Across the two combined areas, edges between individuals (nodes) of different species accounted for 42% of the total weight, but 55% of all links (mean intraspecific degree = 0.42, mean interspecific degree = 0.30). Thus, heterospecifics disproportionately increased the edge density of the network (the number of links), increasing the connections between otherwise disconnected conspecifics. However, the average association strength between heterospecifics was weaker than the average association strength between conspecifics, suggesting that heterospecific associations are less temporally stable. Finally, assortment by degree (where assortment indicates disproportionately strong connections between nodes with similar degrees [32]) was higher in the network combining both types of associations (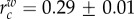) than in the network with only intraspecific links (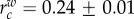).

### (a) Multi-network network-based diffusion analysis

Using a full model-fitting procedure that incorporated both intraspecific and interspecific social networks for each diffusion event, we found that the best-fitting models all included information transfer between conspecifics and heterospecifics (table 2). Over all models, we found that the Akaike weight of models where *s*_intra_ = *s*_inter_ (equal rates of transmission within and between species) accounted for 40.9% of the total weight (table 3). The majority of the support was for different rates of transmission within and between species (total Akaike weight = 59.1%; table 3). Models that included only asocial information acquisition (individual discovery), models with transmission on only one network (either between conspecifics only, or heterospecifics only), and models fitted with homogeneous networks (where all possible network edges = 1) had less than 0.001% of the total combined weight (despite accounting for more than three quarters of all the models tested).

**Table 2.** The top five models ordered by AIC_c_. (All include both the within-species and between-species association networks, and none included the homogeneous network. These models are all additive (i.e. assuming asocial and social learning occur independently) and include a non-constant baseline (i.e. allowing asocial learning rate to increase or decrease over time).)

social transmission within/between species	individual-level variables	d.f.	AIC_c_	Akaike weight (%)
*s*_intra_ ≠ *s*_inter_	species	7	4788.77	56.6
*s*_intra_ = *s*_inter_	species	6	4789.69	35.7
*s*_intra_ = *s*_inter_	residency	5	4793.57	5.1
*s*_intra_ ≠ *s*_inter_	residency	6	4794.98	2.5
*s*_intra_ = *s*_inter_	*none*	4	4804.35	<0.1

**Table 3.** Summary of the total Akaike weight for all models of social transmission and for asocial leaning. (We found strong support models with both intraspecific and interspecific information transfer (*s*_intra_ ≠ 0 and *s*_inter_ ≠ 0). Most of the support was for models where *s*_intra_ ≠ *s*_inter_, but we could not rule out that these might not differ. Further, we found little support for homogeneous spread of information, suggesting that our observed network were a good predictor of information transmission.)

model	network	same (%)	different (%)	intraspecific only (%)	interspecific only (%)
additive	association	40.9	59.1	0	0
	homogeneous	0	0	0	0
multiplicative	association	0	0	0	0
	homogeneous	0	0	0	0
asocial		0^a^			

^a^The total Akaike weight for asocial learning was 2.4 × 10^−27.^

**Figure 3.**
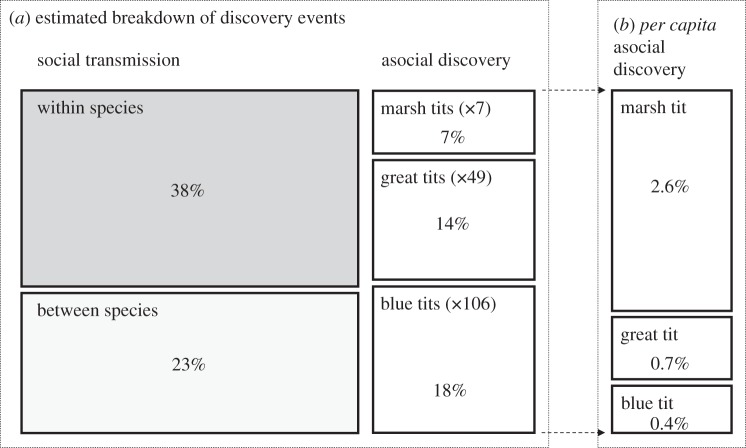
(*a*) Breakdown of discovery events corresponding to the estimated network-based diffusion analysis parameters. The area of each box represents the estimated proportion of individual patch discoveries events (independent arrivals to the patch by each individual) that were a result of transmission within species (38%), transmission between species (23%), or asocial learning (39%). The latter is further broken down by species, with numbers in parentheses giving the observed number of individuals (see table 1). For example, 7% of all arrivals were by marsh tits who discovered the patch without having access to social information. We also calculated the estimated rate of asocial discovery *per capita* (*b*). Each individual marsh tit accounted for 2.6% of all asocial discoveries (totalling 18% of all asocial discoveries by just seven individuals), and thus produced on average 3.7 times more new information than individual great tits and 6.5 times more information than individual blue tits. The size of each boxes represents the estimated percentage of total discoveries (*a*) and asocial discoveries (*b*).

When the rates of social transmission were constrained to be equal between and within species, they were estimated at *s* = 22.2 (95% CI = 6.0–33.9) times the baseline (great tit) rate of asocial learning per unit of network connection, corresponding to an estimated 71% of discoveries being by social transmission. However, in the best-supported model, rates were allowed to differ within and between species. The resulting estimates were *s*_intra_ = 22.2 (95% confidence range 7.5–36.8) and *s*_inter_ = 12.5 (95% confidence range 1.7–25.9), corresponding to 61% of all discoveries being via social transmission (figure 3). The difference *s*_inter_ – *s*_intra_ is therefore estimated to be 9.7 (95% confidence range −6.1 to 23.8), indicating that we have strong evidence of transmission occurring both within and between species. Because individual arrivals were constrained to only count as discovery events if they occurred more than 10 min since the prior arrival, these estimates are also likely to be conservative. Running the best-supported model without this constraint estimates that 73% of all discoveries were social (42% occurring as a result of within-species, and 31% from between-species, social transmission). Full details relating to the estimation of CIs are given in the electronic supplementary material.

The best-supported model estimated that 39% of individuals that first arrived on a food source did so independently of the social network (but see caveat above). An asocial discovery is defined as occurring when a bird discovers a feeder without that location having been transmitted by social transmission from another bird. We found strong support that the rate of asocial discoveries differed between species (total Akaike weight = 92.3%), with marsh tits estimated to be 5.2 times (95% CI: 1.92–10.07) more likely than great tits to find food sources independently of others. Blue tits were estimated to be only 0.8 times as likely as great tits to find food alone (95% CI: 0.27–1.09). At the individual level, each individual marsh tit was estimated to be responsible for 2.6% of all the individual discovery events, much higher than the estimate for individual great tits (0.7%) and blue tits (0.4%; figure 3).

## 4. Discussion

Understanding the relative contribution of different components of social structure is important for the interplay between population structure and population processes. To date, studies of animal groups have often, implicitly or not, assumed that each of the observed links in a social network are formed for the same purpose. Yet different types of associations may be important for different social processes [10]. In this study, we developed a framework that enabled us to quantify how important different relationships, represented as different social networks, are in the diffusion of information. We then tested whether the rates of transmission of social information varied between conspecifics and heterospecifics in a replicated experiment.

After the initial discovery of a food patch, approximately 61% of all individuals that then found the patch were connected to the discoverers through network edges (figure 3). The *s* parameters in our models estimate the rate of transmission per unit of connection for each of the two networks. Because individuals typically had a higher weighted degree (or strength) with conspecifics than heterospecifics, information about food patches is likely to have travelled significantly faster between conspecifics than between species. We found that the rate of information transmission between heterospecifics was generally lower than that between conspecifics, with an estimated transmission rate per unit of connection of 22.2 for intraspecific edges and 12.5 for interspecific edges. However, these results still revealed an important role for interspecific transmission of information in the discovery of new food patches. If individuals did not use heterospecific information, then they would depend on a conspecific having first discovered the resource independently, potentially slowing down the spread of new information through the social network. Interestingly, these results reflect a recent study quantifying local interaction rules for conspecific and heterospecific information-use in the collective behaviour of mixed-species flocks [33].

Associating with, and acquiring information from, different types of individuals may have benefits such as increasing the pool of local knowledge [34,35]. Associations with heterospecifics can also impact the structure of the social network [36], which may positively influence the rate of information spread. For example, the addition of interspecific associations increases network density (connectivity), by adding more edges relative to the total number of nodes, which increases the speed of information flow in networks. However, this increase is dependent on the structure that additional edges produce [37]. The combined mixed-species social network in our study showed a higher level of assortment in network degree than the intraspecific network alone. Assortment by degree occurs when more gregarious individuals are associated more strongly with other gregarious individuals, regardless of species. This type of connectivity creates networks that are robust to fragmentation and promote rapid transmission across the population [38]. Thus, associations between heterospecifics may have a profound influence on the structural properties of the network, with potential fitness consequences (in this case, they influence the spread of information about the location of food resources). Therefore, when estimating consequences of social organization in species that also associate with heterospecifics, it may be important to consider individuals in the context of their entire network, rather than individual species-level networks independently.

There are few studies that have investigated whether removing the opportunity for mixed-species associations influences within-species social processes. The most convincing study to date showed that white-breasted nuthatches (*Sitta carolinensis*) avoided high-risk food resources when tufted titmice (*Baeolophus bicolor*) were removed from flocks [39]. Over time, this led to reduced body condition and an increased rate of over-winter mortality [40]. Similarly, two independent observational studies found that the composition of species on different islands [41] and in different rainforest patches [42] predicted the presence or absence of mixed-species flocking behaviour. Our study highlights the potential importance of heterospecifics in the socioecological landscape of wintering songbirds, by showing that heterospecific associations can lead to an increase in information spread.

Although information moved between heterospecifics, we also found that not all species generated information equally: marsh tits tended to be disproportionately more likely to discover a new patch independently of the network (i.e. asocially). If most individuals learn by social transmission, then the identity of those that make independent discoveries becomes important, since they determine where the information is ‘seeded’ in the group and the subsequent pathway of diffusion. Taken with the evidence that information spreads between species as well as within species, this suggests that marsh tits were the original source of a large amount of social information in our experiments (in total, approx. one-third of discoveries were not the result of information transfer). This may be because marsh tits are more motivated to discover new food sources earlier in the day in order to cache food [26] and, once they discover a new food source, other species parasitize the information they produce [43]. As we control for co-discoveries, our data do not suggest that marsh tits were simply in one mixed-species flock, visited together and simply accessed the feeder first (figure 2). Instead, it is possible that marsh tits may be more likely to find food on their own and actively recruit heterospecifics to new food sites. A recent study of the closely related willow tit (*Poecile montanus*) found that individuals actively recruited heterospecifics to new food sources by making loud contact calls [44]. Similarly marsh tits, which are relatively uncommon, might benefit from dilution of risk or shared vigilance when associating with more numerous heterospecifics. Whether they are parasitized or actively recruit, our study suggests that marsh tits may be a ‘keystone’ species within this mixed-species community during the winter months.

Finally, we have extended the NBDA framework to incorporate multiple candidate networks. This provides a powerful tool for testing competing hypotheses about how information is transferred in multi-dimensional social landscapes. It enables the relative contribution of different potential pathways of social transmission to be quantified. For example, animals form and maintain bonds that may be determined by grooming [45], kinship [46], familiarity [47] or mating networks [48], and these may vary in their importance for different social processes. We then combined this method with a field experiment to show that heterospecifics form an important part of the social landscape in a temperate mixed-species community. We conclude that information was transmitted between those heterospecifics that form mixed-species flocks of birds, and that individuals of different species varied in how much information they produced.

## Supplementary Material

Supplementary Information

## Data accessibility

Supporting data are available from Dryad at http://dx.doi.org/10.5061/dryad.416sp.
