# Supplementary material for: Interspecific social networks promote information transmission in wild songbirds
Source: Proc Biol Sci. 2015 Mar 22;282(1803):20142804. doi: 10.1098/rspb.2014.2804 (PMC4345451; doi:10.1098/rspb.2014.2804)
Supplement: Supplementary Information [file rspb20142804supp1.pdf]

Supplementary Information for

Interspecific social networks promote information transmission in wild songbirds

by W. Hoppitt, D.R. Farine, L.M. Aplin, B.C. Sheldon

Multiple networks NBDA

In the standard NBDA model, the rate at which individual *i* acquires the novel behaviour (e.g. solves a task; discovers a food patch) at time *t* is given by:

$$\lambda_i(t) = \lambda_0(t)\left(s\sum_{j=1}^{N} a_{ij}z_j(t) + 1\right)\left(1 - z_i(t)\right)$$

where $\lambda_0(t)$ is a baseline rate function (see below), a_{ij} is the network connection from *j* to *i*, $z_i(t)$ is the status (0= naïve; 1= informed) of *i* at time *t*, *N* is the number of individuals in the diffusion and *s* is a parameter giving the rate of social transmission (Hoppitt, Boogert & Laland, 2010). We expand the model to include the effects of *M* different networks as follows:

$$\lambda_i(t) = \lambda_0(t)\left(\sum_{k=1}^{M}\left[s_k\sum_{j=1}^{N} a_{ijk}z_j(t)\right] + 1\right)\left(1 - z_i(t)\right)$$

where a_{ijk} is the network connection from *j* to *i* in network *k* and s_k gives the rate of social transmission in network *k*. For the analysis in this paper we have:

$$\lambda_i(t) = \lambda_0(t)\left(s_{intra}\sum_{j=1}^{N} a_{ij,intra}z_j(t) + s_{inter}\sum_{j=1}^{N} a_{ijk,inter}z_j(t) + 1\right)\left(1 - z_i(t)\right)$$

where $a_{ij,intra} = a_{ij}, a_{ij,inter} = 0$ if i and j are the same species and $a_{ij,intra} = 0, a_{ij} = a_{ij}$ otherwise, where a_{ij} is the association network described in the main text.

As with the standard NBDA, the multiple networks NBDA can include other individual-level variables in a multiplicative model:

$$\lambda_i(t) = \lambda_0(t)\left(s_{intra}\sum_{j=1}^{N} a_{ij,intra}z_j(t) + s_{inter}\sum_{j=1}^{N} a_{ijk,inter}z_j(t) + 1\right)exp(LP_i)\left(1 - z_i(t)\right)$$

or an additive model:

$$\lambda_i(t) = \lambda_0(t)\left(s_{intra}\sum_{j=1}^{N} a_{ij,intra}z_j(t) + s_{inter}\sum_{j=1}^{N} a_{ijk,inter}z_j(t) + exp(LP_i)\right)\left(1 - z_i(t)\right)$$

where LP_i is a linear predictor combining V variables analogous to a generalized linear model:

$$LP_i = \sum_{1}^{V} \beta_l x_{li}$$

where x_{li} is the value of the lth individual level variable for individual i, and $exp(\beta_l)$ gives the multiplicative effect of one unit increase in that variable.

There are various version of NBDA: order of acquisition diffusion analysis (OADA, Hoppitt, Boogert & Laland, 2010), discrete time of acquisition diffusion analysis (discrete TADA; Franz & Nunn, 2009), and continuous TADA (Hoppitt, Boogert & Laland, 2010). The multiple networks NBDA could be fitted using any of these methods (see Hoppitt & Laland, 2013 for details of corresponding log-likelihood functions), however, here we used the continuous TADA, as it provides best power and the assumptions seem appropriate in this case (Hoppitt & Laland, 2013). NBDA is easily expanded to multiple independent diffusions, as we do here, by summing the log-likelihood from each diffusion.

In TADA, one can assume a constant baseline rate $\lambda_0(t) = \lambda_0$, or model a specific form for $\lambda_0(t)$. Here we fit models assuming a constant baseline rate, and models allowing that the baseline rate might increase or decrease systematically over the course of a diffusion (Hoppitt, Kandler, Kendal & Laland, 2010). We also fit both additive and multiplicative models for every combination of the individual level variables, "species" (with great tit as the baseline) and "observations", corresponding to the five different hypotheses about social transmission: occurring a) at different rates within and between species; b) at the same rate (s_{within} = $s_{between}$); c) only within species ($s_{between}$= 0); d) only between species (s_{within} = 0); and e) with no social transmission (s_{within} = $s_{between}$= 0). We also fit a complete set of models in which the association network was replaced with a homogeneous network, with all $a_{ij} = 1$, to test whether social transmission was following the association network rather than occurring homogeneously between all individuals in a diffusion (Hoppitt & Laland, 2013). Since diffusions occurred at two different sites, an indicator variable was forced into all models, allowing the relative rate of discovery to differ at each site.

All models were fitted in the R statistical environment version 2.15.2 (R Core Team, 2012), using the optim function.

Supplementary NBDA results

We used an information-theoretic approach using corrected Akaike's Information Criterion (AIC_c) (Burnham & Anderson, 2002). We calculated Akaike weights giving the support for each specific model. The top 5 models are shown in table 2 of the main text.

We obtained model-averaged estimates for all parameters (Burnham & Anderson, 2002). Unfortunately were unable to extract a numerical estimate of the Hessian matrix (Morgan, 2008), so could not estimate standard errors, and therefore were unable to calculate an unconditional standard error to take into account model selection uncertainty (Burnham & Anderson, 2002). Consequently, to quantify the uncertainty in estimates for key parameters, we obtained 95% confidence intervals using the profile likelihood technique, conditional on the best model in which that parameter was present. Given the clear support for the best two models, model selection uncertainty is unlikely to be an important source of uncertainty in parameter estimates. Parameter estimates for social transmission parameters are given in table S1:

Table S1: Estimates of social transmission parameters

Parameter	Model-averaged estimate	95% confidence interval
s *	22.19	5.98 – 33.93
s_{within} $	22.21	7.51 – 36.83
$s_{between}$ $	12.54	1.70 – 25.86
s_{within} - $s_{between}$ $	9.67	-6.14 – 23.81

*From models in which $s = s_{within} = s_{between}$; $ from models in which $s_{within} \neq s_{between}$

s parameters can be interpreted as the rate of discovery by social transmission per unit of association to informed individuals, relative to the baseline rate of asocial learning- i.e. when the linear predictor, LP_i, is set to zero. To give a more intuitive measure of the importance of each type of social transmission, we calculated the probability each event occurred as a result of each type of social transmission (extending the method used by Allen et al., 2013):

$$p_{intra,i} = s_{intra} \sum_{j=1}^{N} a_{ij,intra} z_j(T) \Bigg/ \left(s_{intra} \sum_{j=1}^{N} a_{ij,intra} z_j(T) + s_{inter} \sum_{j=1}^{N} a_{ijk,inter} z_j(T) + exp(LP_i) \right)$$

$$p_{inter,i} = s_{inter} \sum_{j=1}^{N} a_{ij,inter} z_j(T) \Bigg/ \left(s_{intra} \sum_{j=1}^{N} a_{ij,intra} z_j(T) + s_{inter} \sum_{j=1}^{N} a_{ijk,inter} z_j(T) + exp(LP_i) \right)$$

where *i* is the individual discovering the patch and *T* is the time at which discovery occurred. We summed these figures across all discoveries to get an estimate of the number, and hence percentage of events that occurred by each type of social transmission. The same figures were used to calculate the figures quantifying species-specific asocial discovery in figure 1 in the main text.

For individual level variables, we back-transformed the model-averaged estimates and confidence intervals such that they give the multiplicative effect of that variable on discovery rate. Note that because the additive model is overwhelmingly supported over the multiplicative, the implication is that these variables affect only the rate of asocial discovery. The back-transformed estimates are given in table S2:

Table S2: Estimates of effects of individual level variables

Parameter	Total Akaike weight	Model-averaged estimate	95% confidence interval
Blue tit/ great tit	92.3%	0.81	0.27 – 1.09
Marsh tit/ great tit	92.3%	5.20	1.92 – 10.07
Residency (effect of +1 S.D.)	7.7%	1.00	NA*
Cammoor/Higgins	NA$	1.06	NA*

* Since calculating confidence intervals by profile likelihood is a computationally intensive procedure, we only ran them for variable of interest, rather than those included only as potential confounds. $ Site variable forced into model.

Additional references

Burnham, K.P. & Anderson, D.R. (2002) Model selection and Multimodel Inference: A Practical Information-Theoretic Approach. 2nd edition. Springer.

Hoppitt, W. Kandler, A., Kendal, J.R. & Laland, K.N. (2010) The effect of task structure on diffusion dynamics: Implications for diffusion curve and network-based analyses. *Learning & Behavior* **38:** 243-251.

Hoppitt, W. & Laland, K.N. (2013) *Social Learning: An Introduction to Mechanisms, Methods and Models*. Princeton University Press.

Morgan, B.J.T. (2008) *Applied Stochastic Modelling*. 2nd Edition. CRC Press.

R Core Team (2012). R: A language and environment for statistical computing. R Foundation for Statistical Computing, Vienna, Austria. ISBN 3-900051-07-0, URL http://www.R-project.org/.
